# Smoking during pregnancy is associated with child overweight independent of maternal pre-pregnancy BMI and genetic predisposition to adiposity

**DOI:** 10.1038/s41598-022-07122-6

**Published:** 2022-02-24

**Authors:** Theresia M. Schnurr, Lars Ängquist, Ellen Aagaard Nøhr, Torben Hansen, Thorkild I. A. Sørensen, Camilla S. Morgen

**Affiliations:** 1 Novo Nordisk Foundation Center for Basic Metabolic Research, Faculty of Health and Medical Sciences, University of Copenhagen, Blegdamsvej 3B, 2200 Copenhagen, Denmark; 2 Research Unit for Gynecology and Obstetrics, Department of Clinical Research, University of Southern Denmark, Odense, Denmark; 3 Department of Public Health, Section of Epidemiology, Faculty of Health and Medical Sciences, University of Copenhagen, Copenhagen, Denmark; 4 National Institute of Public Health, University of Southern Denmark, Copenhagen, Denmark

**Keywords:** Genetic association study, Genetic variation, Genetic interaction, Epidemiology, Paediatric research, Obesity

## Abstract

High maternal body mass index (BMI) and smoking during pregnancy are risk factors for child overweight. Maternal smoking tends to reduce her BMI and the association of smoking with child overweight may be confounded by or interacting with maternal genetic predisposition to adiposity. In the Danish National Birth Cohort, we investigated whether smoking during pregnancy is associated with child BMI/overweight independent of pre-pregnancy BMI and maternal genetic predisposition to adiposity estimated as total, transmitted and non-transmitted genetic risk scores (GRSs) based on 941 common genetic variants associated with BMI. Smoking during pregnancy was associated with higher child BMI and higher odds of child overweight in a dose–response relationship. The odds ratio (95% CI) for smoking 11 + cigarettes in third trimester versus no smoking was 2.42 (1.30; 4.50), irrespective of maternal BMI and maternal GRSs (total, transmitted or non-transmitted). There were no statistically significant interactions between maternal GRSs and smoking (all p-values for interactions > 0.05). In conclusion, in this study, smoking during pregnancy exhibits a dose–response association with increased child BMI/overweight, independent of maternal pre-pregnancy BMI, maternal transmitted, and non-transmitted genetic predisposition to adiposity. Avoidance of smoking during pregnancy may help prevent childhood obesity irrespective of the mother–child genetic predisposition.

## Introduction

The causes of childhood overweight are complex and poorly understood^1,2^. However, both genetic and environmental factors undoubtedly play a role in its development. Although most of the specific factors remain obscure, maternal smoking during pregnancy appears to be consistently associated with a higher risk of overweight in children^3–7^ and we have previously shown that maternal smoking during pregnancy has a lasting association with child body mass index (BMI), independent of maternal pre-pregnancy BMI and independent of size at birth and growth during infancy^8^.

In view of the well-established strong influence of maternal genetics on both her own BMI and the risk of overweight of her child^9^, it may be speculated that associations between maternal smoking and child overweight are confounded by maternal genetic predisposition to adiposity. This possibility arises if the genetic predisposition of adiposity of the mother increases her tendency to smoke aiming to keep her weight down, and if she continues to do so during pregnancy. In this case, the maternal smoking-associated overweight of the child is partially spurious, with the overweight of the child reflecting the transmission of the genetic predisposition to the child. If the pre-pregnancy smoking had reduced the mother’s pre-pregnancy BMI, then adjustment for it would make the spurious association of maternal smoking with child overweight even stronger. Moreover, it may be questioned whether maternal smoking interacts with her transmitted or non-transmitted genetic predisposition of adiposity or whether the two factors operate independently. Genome-wide association studies (GWASs) in adults indicate that smoking may alter the genetic susceptibility to adiposity^10^. In addition, since maternal socioeconomic position plays an important role as a confounder of the association of maternal smoking and child overweight, the assessment of the associations needs to incorporate that aspect as well^8^.

**Figure 1:**
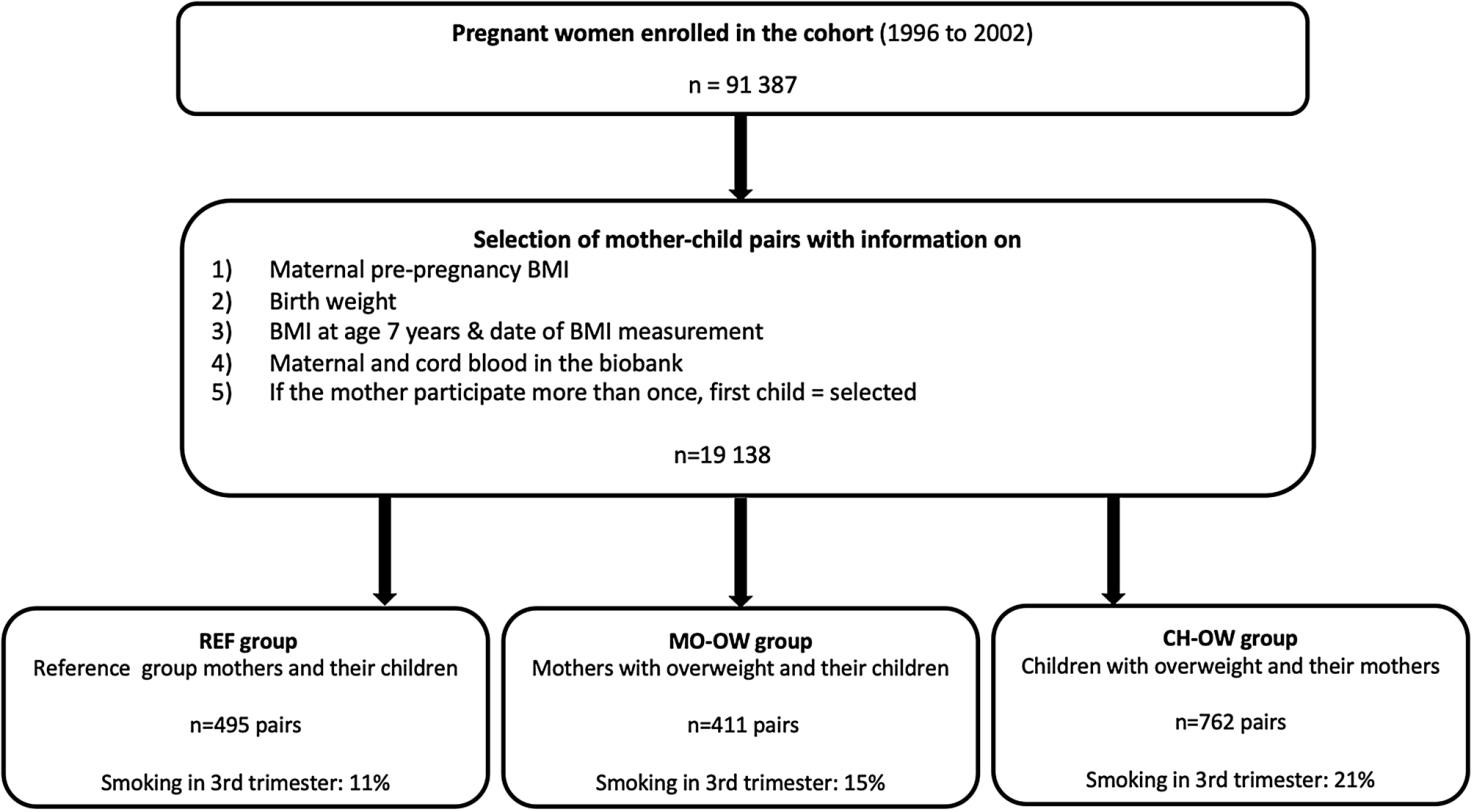
Participant flowchart and selection of mother–child pairs of the REF, MO-OW and CH-OW groups. This study design allowed analyses within the REF group as representative of the subpopulation, of the combined REF and MO-OW groups as an exposure-based cohort design, and the combined REF and CH-OW groups as a case-cohort design. CH-OW: Children with overweight and their mothers (children with overweight group); MO-OW: Mothers with overweight and their children (mothers with overweight group); REF: Randomly selected mothers and their children (reference group).

In this study, we aimed to address whether maternal smoking during pregnancy is associated with child BMI and odds for child overweight independent of maternal pre-pregnancy BMI, maternal genetic predisposition to adiposity, and socioeconomic position. Further, we investigated whether the effect of maternal smoking on child BMI interacts with maternal genetic predisposition to adiposity. Maternal genetic predisposition to adiposity was quantified based on 941 common BMI-associated variants, summarized as total, maternal transmitted and maternal non-transmitted genetic risk scores (GRSs). We employed a general population-based study design within the large Danish National Birth Cohort of ~ 100,000 children, where we compared children of mothers with overweight (the MO-OW group) and children with overweight (the CH-OW group) and to a reference group of children of randomly selected mothers from the same population (the REF group), (Fig. 1). This study design allowed analyses within an exposure-based cohort design (the combined REF and MO-OW groups) and a case-cohort design (the combined REF and CH-OW groups).

## Results

### Descriptive characteristics

The three study samples for the study: a reference group of randomly sampled mother–child pairs (the REF group, n = 495), a group with the most overweight mothers (the MO-OW group, n = 411), and a group with the most overweight children (the CH-OW group, n = 762), see Fig. 1 has, as intended, resulted in large differences in maternal BMI as well as in child BMI and percentage overweight children in the three groups (Table 1). The MO-OW and CH-OW groups had lower socioeconomic position than the REF group, and the CH-OW group had considerably more maternal smoking in pregnancy than the REF group, whereas the MO-OW did not differ from the REF group. Weekly gestational weight gain was slightly lower in the MO-OW than in the REF group, but the CH-OW group did not differ from the REF group in this regard. Maternal age, gestational age at birth and child sex did not differ significantly, but both weight and length of the children at birth were slightly greater in the MO-OW and CH-OW groups than in the REF group. Table S1-S2 provide the distributions of mean pre-pregnancy BMI and the various GRSs by group and by maternal smoking during pregnancy. It appears that there were very small differences by maternal smoking groups barring a somewhat higher BMI of the smoking mothers in the REF group.

**Table 1:** Characteristics of the three groups of mother–child pairs, n = 1668.

	REF group		MO-OW group			CH-OW group		
	n	Values^a^	n	Values^a^	*p* value^b^	n	Values^a^	*p* value^b^
**Maternal pre-pregnancy BMI** (kg/m^2^)	495	22.5 (19.2–39.8)	411	35.0 (33.3–51.3)	< 0.0001	762	24.7 (17.2–33.6)	< 0.0001
**Maternal pre-pregnancy BMI** (z-score)	495	-0.12 ± 0.8	411	3.2 ± 0.8	< 0.0001	762	0.4 ± 0.9	< 0.0001
**Maternal age at birth** (years)	495	30.5 ± 4.1	411	30.1 ± 3.9	0.1	762	30.6 ± 4.2	0.8
**Maternal education/occupational class** (%)	495	100	410	100	< 0.0001	759	100	< 0.0001
Highest level		57.0		39.5			43.6	
Middle level		34.1		44.4			43.6	
Lowest level		8.9		16.2			12.8	
**Smoking in pregnancy** (1st trimester; yes, %)	495	22.1	411	22.0	0.9	759	32.0	< 0.0001
**Smoking in pregnancy** (3rd trimester; yes, %)	461	11.1	391	14.9	0.07	751	21.0	< 0.0001
**Any smoking in pregnancy**^c^	495	22.2	411	22.1	0.9	762	31.9	< 0.0001
**Weekly gestational weight gain** (kg)	408	0.4 ± 0.1	344	0.2 ± 0.2	< 0.0001	616	0.4 ± 0.2	0.6
**Gestational age at birth (**days)	495	281.3 ± 10.1	411	282.0 ± 10.7	0.3	762	281.8 ± 9.5	0.3
**Birth weight** (kg)	495	3.7 (2.1–5.3)	411	3.8 (1.5–5.2)	< 0.0001	762	3.8 (2.4–5.4)	0.001
**Sex** (boys, %)	495	51.1	411	51.1	0.9	762	50.4	0.8
**Length at birth** (cm)	495	52.5 ± 2.1	410	52.8 ± 2.3	0.02	758	53.0 ± 2.2	< 0.0001
**Weight at 7 years** (kg)	495	24.4 (15.0–42.5)	411	26.3 (14.5–50.7)	< 0.0001	762	33.0 (23.2–61.0)	< 0.0001
**Height at 7 years** (cm)	495	126.0 (102.0–141.5)	411	127.0 (106.5–143.0)	< 0.0001	762	130.0 (112.0–146.3)	< 0.0001
**BMI at 7 years** (kg/m^2^)	495	15.5 (10.9–24.5)	411	16.3 (10.1–26.6)	< 0.0001	762	19.7 (17.5–42.4)	< 0.0001
**BMI at 7 years (**z-score)	495	-0.04 ± 1.0	411	0.5 ± 1.1	< 0.0001	762	2.0 ± 0.5	< 0.0001
**Overweight at 7 years** (yes, %)^d^	495	8.5	411	25.3	< 0.0001	762	100.0	< 0.0001

^a^Values are percentages for categorical variables, means ± SD for continuous variables with a normal distribution, or medians (range) for continuous variables with a skewed distribution, ^b^*p* value comparing the MO-OW group with the REF group and the CH-OW group with the REF group, assessed using one-way-ANOVA for continuous variables with a normal distribution, Kruskal–Wallis’s tests for continuous variables with a skewed distribution and Chi-square tests for categorical variables, ^c^Any smoking during pregnancy is a combination of smoking during 1st and 3rd trimester, ^d^Overweight is categorized according to the International Obesity Taskforce reference and obesity is included in the overweight category. *CH-OW* children with overweight and their mothers (children with overweight group), *MO-OW* mothers with overweight and their children (mothers with overweight group), *REF* randomly selected mothers and their children (reference group).

### Associations of maternal smoking and continuous child BMI

Within the REF group, maternal BMI and maternal transmitted GRS were associated with child BMI in both the any smoking and non-smoking groups, whereas the associations of maternal GRS were weaker and absent for the maternal non-transmitted GRS. No statistically significant interactions were found (Table S2, upper panel). Disregarding the possible interactions, the analyses showed that ‘any smoking’ during pregnancy was associated with higher child BMI compared to children of non-smoking mothers (Table 3). This observation was statistically significant in the crude analyses, but became slightly weaker and insignificant when adjusted for maternal BMI and/or GRS (Table 3). The analyses of associations with different levels of smoking in the first and the third trimester showed overall significantly increased child BMI, stronger for smoking more than 10 than for l-10 cigarettes per day, and stronger in the third than in the first trimester. Generally, the adjustments for GRS, maternal BMI and maternal socioeconomic position weakened the associations slightly and made several insignificant, but those for heavy smoking during the third trimester remained the highest and significant (Table 3). Results from analyses with maternal transmitted and maternal non-transmitted GRS as covariate instead of maternal total GRS revealed similar associations and only very small differences, if virtually any, in the estimates (Table S3–S4).

### Associations of maternal smoking and the odds of child overweight

#### Exposure-based cohort design analysis of the MO-OW and REF groups

In the exposure based cohort design, the sample is chosen to include pregnant women with overweight or obesity as the exposure measure of interest. There were no statistically significant interactions of ‘any smoking’ during pregnancy with maternal BMI and the three maternal GRSs (Table 2), but the estimates of the maternal GRS associations with odds of child overweight were all significant (with the exception of maternal non-transmitted GRS among non-smoking mothers), and the estimates were generally higher among smoking than non-smoking mothers. When disregarding possible interactions (Table 4), the analyses showed strong and significant associations of ‘any smoking’ during pregnancy that remained significant irrespective of the adjustments for GRS, maternal BMI and maternal socioeconomic position. Smoking in the first trimester was associated with increased odds of child overweight at both levels of smoking, but higher with smoking more than 10 than 1–10 cigarettes, and the adjustments altered the estimates only slightly. Smoking during the third trimester was generally significantly associated with more than doubling of the odds of child overweight irrespective of the level of smoking and with only minor alterations of the estimates by the adjustments. Similar associations were seen in analyses adjusted for maternally transmitted and non-transmitted GRS instead of maternal total GRS (Table S4–S5).

**Table 2:** Analyses of interactions of maternal smoking during pregnancy with maternal BMI, maternal GRS (total, transmitted and non-transmitted) in the associations with (1) child BMI z-score within the REF group, (2) odds of child overweight (IOTF criteria), and (3) odds of the child being selected into the CH-OW group.

In the REF group (1)	n	No smoking	Any smoking^a^	Interaction
	495	β^2^ (95% CI)	β^2^ (95% CI)	*p* value
Maternal BMI^b^		0.30 (0.17; 0.43)	0.24 (0.02; 0.45)	0.62
Maternal GRS		0.16 (0.06; 0.25)	0.09 (− 0.08; 0.26)	0.43
Maternal transmitted GRS		0.22 (0.12; 0.31)	0.21 (0.05; 0.36)	0.83
Maternal non-transmitted GRS		0.04 (− 0.06; 0.14)	0.03 (− 0.14; 0.20)	0.81

In the MO-OW group and REF group (2)	n	**OR (95% CI)**	**OR (95% CI)**	
	906			
Maternal BMI^b^		1.22 (1.10; 1.34)	1.53 (1.34; 1.76)	0.41
Maternal GRS		1.27 (1.04; 1.56)	1.38 (1.01; 1.89)	0.50
Maternal transmitted GRS		1.39 (1.14; 1.71)	1.62 (1.16; 2.26)	0.60
Maternal non-transmitted GRS		0.96 (0.79; 1.17)	1.51 (1.08; 2.12)	0.18

In the CH-OW group and the REF group (3)	n	**OR**^**4**^** (95% CI)**	**OR**^**4**^** (95% CI)**	
	1257			
Maternal BMI^b^		2.01 (1.76; 2.49)	1.61 (1.17; 2.20)	0.08
Maternal GRS		1.18 (1.04; 1.34)	1.09 (0.83; 1.42)	0.50
Maternal transmitted GRS		1.32 (1.15; 1.50)	1.31 (1.01; 1.69)	0.60
Maternal non-transmitted GRS		1.02 (0.90; 1.16)	0.89 (0.65; 1.13)	0.23

^a^Any smoking during pregnancy is a combination of smoking during 1st and 3rd trimester, ^b^All measures are z-scores.

*CH-OW* children with overweight and their mothers (children with overweight group), *MO-OW* mothers with overweight and their children (mothers with overweight group), *REF* randomly selected mothers and their children (reference group).

#### Case-cohort design analysis of the CH-OW and REF groups

In the analyses based on a case-cohort design, we have used a sub-cohort of the DNBC with children with overweight or obesity and a random sample (the REF group) to increase the statistical power. The outcome of interest is odds for being sampled/belonging to the CH-OW group. The maternal BMI and maternal transmitted GRS were associated with being in the CH-OW group, whereas the associations were weaker for the maternal GRS and absent for the maternal non-transmitted GRS. The analyses of interactions of ‘any smoking’ during pregnancy and maternal BMI and with the three maternal GRSs were not significant (Table 2). Disregarding possible interactions in the analyses. ‘Any smoking’ during pregnancy was consistently and significantly associated with odds of belonging to the CH-OW group irrespective of the adjustments for GRS, maternal BMI and maternal socioeconomic position (Table 5). Smoking during the first trimester was generally associated with greater odds of belonging to the CH-OW group than for no smoking, but only significant for smoking more than 10 cigarettes per day, and the adjustments reduced the estimates only slightly. Smoking during the third trimester was also generally associated with significantly increased odds, at higher levels than for smoking during the first trimester, and higher for smoking more than 10 than 1–10 cigarettes per day. Similar associations were seen in analyses adjusted for maternally transmitted and non-transmitted GRS instead of maternal total GRS (Table S7–S8).

## Discussion

We examined whether maternal smoking during pregnancy is associated with child BMI and odds for overweight independent of maternal pre-pregnancy BMI, maternal genetic predisposition to adiposity, transmitted or non-transmitted, and socioeconomic position. We found that maternal smoking significantly is associated with a higher child BMI and increased odds for overweight at age 7, independent of these adjustments. We explored whether maternal smoking during pregnancy interacts with maternal genetic predisposition for adiposity, whether transmitted to the child or not, on the effect on child BMI and odds for childhood overweight, and we found no indications of any substantial interactions.

An important strength of the study is the unique design, which makes it possible to examine gene-smoking interactions in three different samples of mother–child pairs, characterized with very different distributions of BMI. Due to this selection, we were able to apply an exposure-based cohort design and a case-cohort design within the large Danish National Birth Cohort, harvesting a considerable part of the statistical power of what might otherwise be obtained by full cohort analyses as demonstrated by the precision of the estimates despite the relatively small sample size^11^. Second, the partition of the maternal genetic predisposition to adiposity into transmitted versus non-transmitted GRSs made it possible to distinguish the effect of the total maternal GRS into the BMI-increasing risk alleles that the mother transmitted to her child and the BMI-increasing risk alleles that the mother carries but did not pass on to her child. Further strengths are the long follow-up, which allowed us to examine whether possible effects are lasting through age 7 years.

Various limitations of our study need to be considered. There is an element of selection into the cohort and loss to follow-up, which may be skewed and create bias. Firstly, the generalisability of the results may be limited since women with a higher social position are overrepresented in the Danish National Birth Cohort^12^. The prevalence of overweight and obesity among the children is lower than in the general Danish population, as seen, for example, in the REF group and in the full cohort^11^. Although this is a general finding in the population, it could here also be a result of the parent-reported 7-years weight, which may be underreported due to several reasons. The literature on the accuracy on self-reported health behaviours suggests that although most people report accurately, the respondents tend to underreport characteristics that are considered to be undesirable or negative such as extremes of body height and weight^13^. Furthermore, reporting height and weight of others is less accurate and less reliable than self-reports^14^, so there may be greater random and possibly also systematic errors in the child’s weight and height as reported by a parent. The height and weight information of the child at 7 years have been validated in an independent study within the Danish National Birth Cohort and, importantly, the validation showed no trends towards increasing differences in weight or height with increasing corresponding averages, suggesting that the disagreements may be treated as random errors^15^. The percentage of women who reported that they had been smoking during pregnancy was quite high^16^. On average, more than 26% of the women had been smoking during pregnancy across the three samples (22% in the REF group, 23% in the MO-OW group and 32% in the CH-OW group), which corresponds with the overall smoking prevalence of 26% in the entire Danish National Birth Cohort and is at the same level or a little bit lower than among Danish women at that time^8,16,17^. It is difficult to determine how various potential under- or over-reporting would affect the estimates, but if self-reports on child BMI, maternal BMI and smoking are all underreported, the effect of the interaction may be biased in an unpredictable way or predictable only under untestable assumptions.

The observation that maternal smoking during pregnancy is associated with childhood overweight, independent of maternal BMI or genetic predisposition to adiposity speaks against the contention that the association of smoking during pregnancy with child overweight is spuriously driven by a tendency of overweight mothers to keep their body weight down by smoking^18^. Furthermore, we do not see an interaction of maternal genetic predisposition with maternal smoking on child overweight.

Genetic factors account for 40–70% of the within-population variance in human adiposity based on multiple family, twin and adoption studies^1,19,20^. During the past decade, GWASs have identified several loci associated to different measures of adiposity in adults and in children. Altogether, these 941 genetic variants included in the present study explain about 6% of the genetic variation in adult BMI^9^. Consequently, yet unidentified additional molecular genetic variation may still be important for the smoking effects^9,21^. Smoking may alter the genetic susceptibility to adiposity^10^ as demonstrated by a large meta-analysis of GWASs for adiposity-related traits that adjusted for smoking behaviour in the statistical model. The meta-analysis included 51,080 current smokers and 190,178 non-smokers of mainly European ancestry and 23 novel loci for BMI and central adiposity were identified. That study also identified 9 loci with convincing evidence of gene-smoking interaction on adiposity-related traits, highlighting novel biological functions that included response to oxidative stress, addictive behaviour, and regulatory functions.

Epigenetic factors may also play a role in our observed association that maternal smoking during pregnancy is associated with childhood overweight. A large meta-analysis of methylation data showed that several thousand CpG sites on the DNA of the new-borns were differentially methylated by maternal smoking^22^. On the other hand, there is little evidence to suggest that the methylation of the DNA is associated with child overweight^23^. However, the genetic profile influences the epigenetic profile^24^. so future studies are warranted to follow-up on our findings focusing on possible genetic-epigenetic interactions.

In conclusion, our study suggests that the effect of maternal smoking during pregnancy is not confounded by and does not interact with maternal pre-pregnancy BMI, maternal total, transmitted or non-transmitted, genetic predisposition to adiposity on child BMI or odds for child overweight. While smoking by itself is associated with increased BMI and increased odds of overweight of the child, we found that this association is independent of pre-pregnancy BMI, maternal genetic predisposition to adiposity and socioeconomic position.

## Methods

### Study population

The Danish National Birth Cohort served as the basis for the study. The cohort was established during the years 1996–2002, where a total of 100,413 pregnancies among 92,274 women were enrolled into the cohort from all over Denmark^25^. The women gave detailed information during computer-assisted telephone interviews around gestational weeks 16 and 30, and around 6 and 18 months after birth. Reports on weight and height of the children originates from a web-based follow-up, conducted when the children were around 7 years old. The Danish National Birth Cohort biobank stored blood samples collected from the mothers during pregnancy and from cord blood at birth.

As shown in the flow-chart (Fig. 1), we identified a subpopulation within the Danish National Birth Cohort with available information on maternal pre-pregnancy BMI, birth weight, BMI when the children were 7 years old, and blood samples from the mothers and their children. If mothers participated in the Danish National Birth Cohort with several pregnancies/children, only her first child was considered for genotyping. In total, 19,138 mother–child pairs fulfilled these criteria. According to the study design, a total of 1,668 mother–child pairs were genotyped and included in the study.

### Study design

We selected three study samples for genome-wide genotyping from this subpopulation: a reference group of randomly sampled mother–child pairs (the REF group, n = 495), a group with the most overweight mothers (the MO-OW group, n = 411), and a group with the most overweight children (the CH-OW group, n = 762), see Fig. 1. This study design allowed analyses within the REF group as representative of the subpopulation, of the combined REF and MO-OW groups as an exposure-based cohort design, and the combined REF and CH-OW groups as a case-cohort design.

In the exposure based cohort design, the sample is chosen to include pregnant women with overweight or obesity as the exposure measure of interest. In the case-cohort design, we have combined a case sample of children with overweight or obesity and a random sample (the REF group) for comparison. The outcome of interest in these analyses is odds for being sampled/belonging to the CH-OW group. The case-cohort design increases the statistical power which can be seen in the relatively narrow confidence intervals even for a smaller sample as we have in this study^26^.

### Exposure and outcome measures

Maternal pre-pregnancy height and weight and information on maternal socioeconomic position (ranked, based on a combination of education and occupation)^27^ and parity, were obtained from the first interview in gestational week 16.

Information on height and weight is based parent reports. The children were measured by the parents for 67% of the children. For the remaining part, the measurements were objective measurements taken by the school doctor, public health nurse, or the general practitioner and reported in a “child’s book” which is kept by the parents. At the interview these measures were reported by the parents^28^.

The women reported on smoking during pregnancy at the first and second pregnancy interview (first and third trimester, respectively, categorised as: no smoking, smoking of 1–10 cigarettes per day and smoking of 11 + cigarettes per day).

### Genotyping and GRS calculation

The genotyping procedure using Illumina Human Beadchip technologies (Illumina, San Diego, CA, USA), the genotype quality control, and genotype imputations to the Haplotype Reference Consortium (HRC, release 1) are described in detail elsewhere^29^. For the construction of GRSs, we combined the 941 genetic variants that were associated with BMI at a revised genome-wide significance threshold (*P* < 1 × 10^–8^) in the large meta-analysis of GWASs, including ~ 700,000 adults of European ancestry, conducted by Yengo et al.^9^. We generated weighted BMI-increasing GRSs by summing the genotype dosages of the BMI-increasing alleles weighted by the effect sizes of the variants. Using a previously described method that examines haplotypes to estimate allelic transmission^30^ we derived the maternal transmitted and maternal non-transmitted allele for each of the 941 BMI-associated genetic variants using the public available code on GitHub: https://github.com/rnbeaumont/poe_generator. We then constructed weighted child, maternal transmitted and maternal non-transmitted BMI-increasing GRSs as described and validated in our previous work^29^.

### Statistical analyses

#### Variable transformations

BMI was calculated as weight (kg)/[height (m)]^2^. We transformed maternal BMI to internal sex-specific z-scores, based on an internal reference constituted by the entire cohort with information on BMI (n ~ 90 000). Child BMI was converted to sex- and age-specific z-scores by the Lambda-Mu-Sigma (LMS) method^31,32^. Child overweight at age 7 years was defined according to the International Obesity Task Force (IOTF) reference. Based on age and sex specific cut off points from 2 to 18 years children are categorized as having either a normal weight, overweight or obesity^33^. We made a binary variable for smoking during pregnancy [‘any smoking’ (yes/no)] by a combination of the information on smoking from both the first and the third trimester interviews. None of the women had missing information on smoking both in the first and the third trimester. For the few mothers with information from one interview only, that information was used (total number with information from one trimester only, n = 72 corresponding to 4.3%). For descriptive purposes, we rescaled the GRSs to reflect the number of BMI-increasing alleles carried by each mother or child using a previously described method^34^. In the statistical analyses, we used GRSs standardized into z-scores.

#### Descriptive group comparisons

The mean maternal pre-pregnancy BMIs according to smoking are presented in Table S1. We tested for differences in characteristics in the MO-OW group *versus* the REF group and in the CH-OW group *versus* the REF group by t-tests for continuous variables with a normal-like distribution, Mann–Whitney rank sum tests for continuous variables with a skewed distribution, and Chi-square tests for categorical variables (Table 1; Table S2).

#### Statistical modelling

Using regression models with two-way interaction terms, we first investigated, if there were appreciable statistical interactions between the variable ‘any smoking’ and the maternal pre-pregnancy BMI or the maternal GRSs (total, transmitted and non-transmitted) in their associations with the outcome variables, child BMI or child overweight (Table 2). When statistical interactions were absent or weak, we estimated the main effects of the ‘any smoking’ variable and the three-level smoking variables in the first and the third trimester on the outcome variables in a series of five regression models. These models included the following maternal covariates: (1) none, (2) BMI, (3) total GRS, (4) BMI and total GRS, and (5) BMI, total GRS and socioeconomic position (Tables 3, 4, 5). The analyses were repeated where the maternal total GRS was replaced by maternal transmitted GRS and maternal non-transmitted GRS (Tables S3–S8).

**Table 3:** Smoking during pregnancy and child BMI at age 7 years, in the randomly selected REF group.

	n	Crude analysis	Adjusted^a^	Adjusted^b^	Adjusted^c^	Adjusted^d^
		β (95% CI)	β (95% CI)	β (95% CI)	β (95% CI)	β (95% CI)
**Any smoking during pregnancy**^**e**^	495					
No	385	0.00	0.00	0.00	0.00	0.00
Yes	110	0.23 (0.03; 0.44)	0.18 (-0.02; 0.38)	0.22 (0.02; 0.42)	0.17 (-0.02; 0.37)	0.16 (-0.04; 0.36)
**Smoking 1**^**st**^** trimester**	494					
No smoking	385	0.00	0.00	0.00	0.00	0.00
1–10 cigarettes per day	68	0.16 (-0.09; 0.40)	0.12 (-0.12; 0.36)	0.15 (-0.09; 0.40)	0.12 (-0.12; 0.36)	0.11 (-0.13; 0.35)
11 + cigarettes per day	41	0.38 (0.07; 0.68)	0.30 (0.00; 0.61)	0.34 (0.04; 0.65)	0.28 (-0.02; 0.58)	0.27 (-0.03; 0.57)
**Smoking 3**^**rd**^** trimester**	461					
No smoking	410	0.00	0.00	0.00	0.00	0.00
1–10 cigarettes per day	27	0.30 (-0.08; 0.67)	0.26 (-0.11; 0.63)	0.29 (-0.08; 0.66)	0.25 (-0.11; 0.61)	0.23 (-0.14; 0.59)
11 + cigarettes per day	24	0.63 (0.23; 1.02)	0.57 (0.18; 0.96)	0.62 (0.23; 1.01)	0.57 (0.19; 0.96)	0.56 (0.16; 0.95)

^a^Adjusted for maternal BMI z-score, ^b^Adjusted for maternal total GRS, ^c^Adjusted for maternal BMI and total GRS, ^d^Adjusted for maternal BMI, total GRS and socioeconomic position, ^e^Any smoking during pregnancy is a combination of smoking during 1st and 3rd trimester. The numbers (n) in the adjusted analyses are slightly smaller due to missing values on covariates. *REF* randomly selected mothers and their children (reference group).

**Table 4:** Smoking during pregnancy and odds of child overweight (IOTF criteria) at age 7 years, in the combined reference group (REF) and groups of mothers with overweight and their children (MO-OW).

	n	Crude analysis	Adjusted^a^	Adjusted^b^	Adjusted^c^	Adjusted^d^
		OR (95% CI)	OR (95% CI)	OR (95% CI)	OR (95% CI)	OR (95% CI)
**Any smoking during pregnancy**^**e**^	904					
No	705	1.00	1.00	1.00	1.00	1.00
Yes	201	2.04 (1.39; 2.99)	2.03 (1.36; 3.03)	2.03 (1.38; 2.99)	2.04 (1.37; 3.04)	1.99 (1.33; 2.99)
**Smoking 1st trimester**	904					
No smoking	705	1.00	1.00	1.00	1.00	1.00
1–10 cigarettes per day	109	1.69 (1.02; 2.08)	1.82 (1.07; 3.09)	1.71 (1.03; 2.86)	1.84 (1.08; 3.13)	1.79 (1.05; 3.06)
11 + cigarettes per day	90	2.62 (1.59; 4.31)	2.40 (1.43; 4.02)	2.57 (1.56; 4.24)	2.40 (1.43; 4.01)	2.37 (1.41; 4.01)
**Smoking 3rd trimester**	850					
No smoking	741	1.00	1.00	1.00	1.00	1.00
1–10 cigarettes per day	50	2.68 (1.41; 5.07)	2.68 (1.38; 5.21)	2.57 (1.35; 4.90)	2.61 (1.34; 5.09)	2.49 (1.27; 4.99)
11 + cigarettes per day	59	2.68 (1.48; 4.83)	2.42 (1.32; 4.44)	2.70 (1.49; 4.89)	2.45 (1.34; 4.50)	2.42 (1.30; 4.50)

^a^Adjusted for maternal BMI z-score, ^b^Adjusted for maternal total GRS, ^c^Adjusted for maternal BMI and total GRS, ^d^ Adjusted for maternal BMI, total GRS and socioeconomic position, ^e^Any smoking during pregnancy is a combination of smoking during 1st and 3rd trimester. The numbers (n) in the adjusted analyses are slightly smaller due to missing values on covariates. *REF* randomly selected mothers and their children (reference group), *MO-OW* mothers with overweight and their children (mothers with overweight group).

**Table 5:** Smoking during pregnancy and odds of children being sampled into the group with overweight (CH-OW), based on the combined REF group and the CH-OW group.

	n	Crude analysis	Adjusted^a^	Adjusted^b^	Adjusted^c^	Adjusted^d^
		OR (95% CI)	OR (95% CI)	OR (95% CI)	OR (95% CI)	OR (95% CI)
**Any smoking during pregnancy**^**e**^	1257					
No	904	1.00	1.00	1.00	1.00	1.00
Yes	353	1.66 (1.28; 2.15)	1.59 (1.22; 2.08)	1.66 (1.28; 2.16)	1.59 (1.22; 2.09)	1.48 (1.12; 1.95)
**Smoking 1st trimester**	1253					
No smoking	901	1.00	1.00	1.00	1.00	1.00
1–10 cigarettes per day	185	1.30 (0.94; 1.80)	1.26 (0.90; 1.77)	1.30 (0.94; 1.81)	1.27 (0.90; 1.78)	1.17 (0.83; 1.65)
11 + cigarettes per day	167	2.32 (1.59; 3.38)	2.20 (1.51; 3.27)	2.31 (1.59; 3.38)	2.22 (1.51; 3.27)	2.09 (1.41; 3.10)
**Smoking 3rd trimester**	1212					
No smoking	1003	1.00	1.00	1.00	1.00	1.00
1–10 cigarettes per day	90	1.63 (1.02; 2.60)	1.52 (0.94; 2.47)	1.61 (1.01; 2.57)	1.52 (0.93; 2.46)	1.39 (0.86; 2.27)
11 + cigarettes per day	119	2.73 (1.74; 4.40)	2.80 (1.74; 4.53)	2.82 (1.77; 4.49)	2.82 (1.75; 4.55)	2.59 (1.58; 4.24)

^a^Adjusted for maternal BMI z-score, ^b^Adjusted for maternal total GRS, ^c^Adjusted for maternal BMI and total GRS, ^d^Adjusted for maternal BMI, total GRS and socioeconomic position, ^e^Any smoking during pregnancy is a combination of smoking during 1st and 3rd trimester. The numbers (n) in the adjusted analyses are slightly smaller due to missing values on covariates. *CH-OW* children with overweight and their mothers (children with overweight group), *REF* randomly selected mothers and their children (reference group).

Within the reference group, we assessed the associations with child BMI using linear regression analyses (Table 3; Tables S3–S4). In combined analyses of the exposure-based cohort design with the MO-OW and REF groups, we used logistic regression analyses to assess the associations with child overweight defined by the IOTF criteria as the outcome (Table 4; Tables S5–S6). In the combined analyses of the case-cohort design with the CH-OW and REF groups, we used logistic regression analyses to estimate the odds of being in the CH-OW group (Table 5; Tables S7–S8).

### Ethical statement

The women gave written informed consent at enrolment*.* The establishment of the cohort was approved under Ref. No (KF) 01-471/94 by the Committee on Biomedical Research Ethics. The Danish Data Protection Agency approved the data collection of the cohort (j.nr. 2012-54-0268 until September 2015 and by j.nr. 2015-57-0102 after this date), the 7-year follow-up (Case No. 2004-41-4078). The study procedures were in accordance with the principles of the Declaration of Helsinki.

## Supplementary Information

Supplementary Information.

## Data Availability

Relevant data for the present study are within the paper and its Supporting Information files. Access to additional individual data underlying the findings may be approved with some restrictions. Data is available from the Danish National Birth Cohort and can be requested through the steering committee of the study who can be contacted under dnbc-research@ssi.dk. More information regarding access to data can be found on the Danish National Birth Cohort website http://www.dnbc.dk/access-to-dnbc-data.
