# Supplementary material for: Smoking during pregnancy is associated with child overweight independent of maternal pre-pregnancy BMI and genetic predisposition to adiposity
Source: Sci Rep. 2022 Feb 24;12:3135. doi: 10.1038/s41598-022-07122-6 (PMC8873398; doi:10.1038/s41598-022-07122-6)
Supplement: Supplementary file 1 — Supplementary Information. [file 41598_2022_7122_MOESM1_ESM.docx]

**Smoking during pregnancy is associated with child overweight independent of maternal pre-pregnancy BMI and genetic predisposition to adiposity**

Theresia M. Schnurr^1^, Lars Ängquist^1^, Ellen Aagaard Nohr^2^, Torben Hansen^1^, Thorkild I.A Sørensen^1,3^, Camilla S. Morgen^1,3,4^

^1^ Novo Nordisk Foundation Center for Basic Metabolic Research, Faculty of Health and Medical Sciences, University of Copenhagen, Denmark.

^2^ Research Unit for Gynecology and Obstetrics, Department of Clinical Research, University of Southern Denmark

^3^ Department of Public Health, Section of Epidemiology, Faculty of Health and Medical Sciences, University of Copenhagen, Denmark.

^4^ National Institute of Public Health, University of Southern Denmark, Copenhagen, Denmark.

**Table S1. Maternal pre-pregnancy BMI in the 3 mother-child pairs according to smoking.**

|  |  | **REF group** |  |  | **MO-OW group** |  |  | **CH-OW group** |
| --- | --- | --- | --- | --- | --- | --- | --- | --- |
|  | **n** | Mean ± SD |  | **n** | Mean ± SD |  | **n** | Mean ± SD |
| **Any smoking during pregnancy^1^** | 495 |  |  | 413 |  |  | 762 |  |
| No | 385 | 21.9 ± 2.2 |  | 320 | 37.0 ± 3.2 |  | 519 | 25.1 ± 3.7 |
| Yes | 110 | 23.7 ± 3.5 |  | 93 | 37.3 ± 3.7 |  | 243 | 25.0 ± 3.8 |
|  |  |  |  |  |  |  |  |  |
| **Smoking 1^st^ trimester** | 494 |  |  | 412 |  |  | 759 |  |
| No smoking | 385 | 21.9 ± 2.2 |  | 320 | 37.0 ± 3.2 |  | 516 | 25.2 ± 3.7 |
| 1-10 cigarettes per day | 68 | 23.5 ± 3.6 |  | 41 | 37.6 ± 4.0 |  | 117 | 25.0 ± 3.6 |
| 11+ cigarettes per day | 41 | 24.1 ± 3.3 |  | 51 | 37.0 ± 3.4 |  | 126 | 25.2 ± 3.9 |
|  |  |  |  |  |  |  |  |  |
| **Smoking 3^rd^ trimester** | 461 |  |  | 391 |  |  | 751 |  |
| No smoking | 410 | 23.0 ± 3.2 |  | 332 | 37.1 ± 3.2 |  | 593 | 25.1 ± 3.6 |
| 1-10 cigarettes per day | 27 | 23.6 ± 3.5 |  | 23 | 37.5 ± 3.7 |  | 63 | 25.5 ±3.9 |
| 11+ cigarettes per day | 24 | 23.9 ± 4.1 |  | 36 | 36.5 ± 2.7 |  | 95 | 24.9 ±4.2 |

^1.^ Any smoking during pregnancy is a combination of smoking during 1^st^ and 3^rd^ trimester.

CH-OW: Children with overweight and their mothers (children with overweight group); MO-OW: Mothers with overweight and their children (mothers with overweight group); REF: Randomly selected mothers and their children (reference group).

**Table S2. Various GRSs comprised of 941 BMI-associated common genetic variants according to smoking during pregnancy in the three groups of children with available GWAS.**

|  |  | **REF group** |  |  | **MO-OW group** |  |  |  | **CH-OW group** |  |  |
| --- | --- | --- | --- | --- | --- | --- | --- | --- | --- | --- | --- |
|  | **n** | Mean ± SD |  | **n** | Mean ± SD |  | **n** |  | Mean ± SD |  | p-value |
| **Child GRS** | 495 | 901.9 ± 18.2 |  | 411 | 908.0 ± 18.1 |  | 762 |  | 908.0 ± 18.3 |  | 0.84 |
| No smoking | 385 | 901.5 ± 17.8 |  | 320 | 908.7 ± 17.8 |  | 519 |  | 908.2 ±18.0 |  |  |
| Any smoking^1^ | 110 | 903.4 ± 19.7 |  | 91 | 905.9 ± 19.0 |  | 243 |  | 907.6 ±19.6 |  |  |
|  |  |  |  |  |  |  |  |  |  |  |  |
| **Maternal GRS** | 495 | 902.7 ± 17.4 |  | 411 | 910.8 ± 17.9 |  | 762 |  | 905.3 ± 18.0 |  | 0.30 |
| No smoking | 385 | 902.3 ± 17.1 |  | 320 | 910.8 ± 17.9 |  | 519 |  | 905.5 ± 18.1 |  |  |
| Any smoking | 110 | 904.4 ± 18.3 |  | 91 | 909.6 ± 18.9 |  | 243 |  | 904.4 ± 18.2 |  |  |
|  |  |  |  |  |  |  |  |  |  |  |  |
| **Maternal transmitted GRS** | 495 | 444.5 ± 12.2 |  | 411 | 448.5 ± 12.1 |  | 762 |  | 447.8 ± 12.4 |  | 0.65 |
| No smoking | 385 | 444.3 ± 11.8 |  | 320 | 448.6 ±11.8 |  | 519 |  | 447.7 ± 12.2 |  |  |
| Any smoking | 110 | 445.2 ± 13.6 |  | 91 | 448.2 ± 12.9 |  | 243 |  | 448.1 ± 13.2 |  |  |
|  |  |  |  |  |  |  |  |  |  |  |  |
| **Maternal non-transmitted GRS** | 495 | 448.8 ± 12.5 |  | 411 | 451.0 ± 12.5 |  | 762 |  | 446.6 ± 12.5 |  | 0.19 |
| No smoking | 385 | 446.5 ± 12.4 |  | 320 | 450.9 ± 12.5 |  | 519 |  | 446.8 ± 12.5 |  |  |
| Any smoking | 110 | 448.0 ± 12.9 |  | 91 | 451.5 ± 12.8 |  | 243 |  | 446.0 ± 12.6 |  |  |

^1.^ Any smoking during pregnancy is a combination of smoking during 1^st^ and 3^rd^ trimester.

CH-OW: Children with overweight and their mothers (children with overweight group); MO-OW: Mothers with overweight and their children (mothers with overweight group); REF: Randomly selected mothers and their children (reference group).

**Table S3. Smoking during pregnancy and child BMI at age 7 years, adjusted for maternal transmitted GRS, in the randomly selected REF group.**

|  | |  | Crude analysis | Adjusted^1^ | Adjusted^2^ | Adjusted^3^ | Adjusted^4^ |
| --- | --- | --- | --- | --- | --- | --- | --- |
|  | | **n** | β (95% CI) | β (95% CI) | β (95% CI) | β (95% CI) | β (95% CI) |
| **Any smoking during pregnancy^5^** | | 495 |  |  |  |  |  |
| No | | 385 | 0.00 | 0.00 | 0.00 | 0.00 | 0.00 |
| Yes | | 110 | 0.23 (0.03; 0.44) | 0.18 (-0.02; 0.38) | 0.22 (0.02; 0.42) | 0.17 (-0.02; 0.37) | 0.16 (-0.04; 0.35) |
|  | |  |  |  |  |  |  |
| **Smoking 1^st^ trimester** | | 494 |  |  |  |  |  |
| No smoking | | 385 | 0.00 | 0.00 | 0.00 | 0.00 | 0.00 |
| 1-10 cigarettes per day | | 68 | 0.16 (-0.09; 0.40) | 0.12 (-0.12; 0.36) | 0.17 (-0.07; 0.41) | 0.13 (-0.10; 0.37) | 0.12 (-0.12; 0.36) |
| 11+ cigarettes per day | | 41 | 0.38 (0.07; 0.68) | 0.30 (0.00; 0.61) | 0.31 (0.01; 0.61) | 0.25 (-0.41; 0.55) | 0.24 (-0.06; 0.54) |
|  | |  |  |  |  |  |  |
| **Smoking 3^rd^ trimester** | | 461 |  |  |  |  |  |
| No smoking | | 410 | 0.00 | 0.00 | 0.00 | 0.00 | 0.00 |
| 1-10 cigarettes per day | | 27 | 0.30 (-0.08; 0.67) | 0.26 (-0.11; 0.63) | 0.30 (-0.06; 0.67) | 0.26 (-0.09; 0.62) | 0.24 (-0.12; 0.60) |
| 11+ cigarettes per day | | 24 | 0.63 (0.23; 1.02) | 0.57 (0.18; 0.96) | 0.60 (0.21; 0.99) | 0.55 (0.17; 0.93) | 0.53 (0.15; 0.92) |
|  | 1. Adjusted for maternal BMI z-score, 2. Adjusted for maternal transmitted GRS, 3. Adjusted for maternal BMI and transmitted GRS, 4. Adjusted for maternal BMI, transmitted GRS and socioeconomic position, 5. Any smoking during pregnancy is a combination of smoking during 1^st^ and 3rd trimester. The numbers (n) in the adjusted analyses are slightly smaller due to missing values on covariates. REF: Randomly selected mothers and their children (reference group). | | | | | | |

**Table S4. Smoking during pregnancy and child BMI at age 7 years, adjusted for maternal non-transmitted GRS, in the randomly selected REF group.**

|  | |  | Crude analysis | Adjusted^1^ | Adjusted^2^ | Adjusted^3^ | Adjusted^4^ | |
| --- | --- | --- | --- | --- | --- | --- | --- | --- |
|  | | **n** | β (95% CI) | β (95% CI) | β (95% CI) | β (95% CI) | β (95% CI) | |
| **Any smoking during pregnancy^5^** | | 495 |  |  |  |  |  | |
| No | | 385 | 0.00 | 0.00 | 0.00 | 0.00 | 0.00 | |
| Yes | | 110 | 0.23 (0.03; 0.44) | 0.18 (-0.02; 0.38) | 0.23 (0.03; 0.42) | 0.18 (-0.02; 0.38) | 0.16 (-0.04; 0.36) | |
|  | |  |  |  |  |  |  | |
| **Smoking 1^st^ trimester** | | 494 |  |  |  |  |  | |
| No smoking | | 385 | 0.00 | 0.00 | 0.00 | 0.00 | 0.00 | |
| 1-10 cigarettes per day | | 68 | 0.16 (-0.09; 0.40) | 0.12 (-0.12; 0.36) | 0.15 (-0.09; 0.40) | 0.12 (-0.12; 0.36) | 0.11 (-0.14; 0.35) | |
| 11+ cigarettes per day | | 41 | 0.38 (0.07; 0.68) | 0.30 (0.00; 0.61) | 0.38 (0.07; 0.68) | 0.30 (0.00; 0.60) | 0.29 (-0.02; 0.59) | |
|  | |  |  |  |  |  |  | |
| **Smoking 3^rd^ trimester** | | 461 |  |  |  |  |  | |
| No smoking | | 410 | 0.00 | 0.00 | 0.00 | 0.00 | 0.00 | |
| 1-10 cigarettes per day | | 27 | 0.30 (-0.08; 0.67) | 0.26 (-0.11; 0.63) | 0.28 (-0.10; 0.65) | 0.26 (-0.09; 0.62) | 0.23 (-0.14; 0.60) | |
| 11+ cigarettes per day | | 24 | 0.63 (0.23; 1.02) | 0.57 (0.18; 0.96) | 0.64 (0.24; 1.03) | 0.55 (0.17; 0.93) | 0.56 (0.16; 0.95) | |
|  | 1. Adjusted for maternal BMI z-score, 2. Adjusted for maternal non-transmitted GRS, 3. Adjusted for maternal BMI and non-transmitted GRS, 4. Adjusted for maternal BMI, non-transmitted GRS and socioeconomic position, 5. Any smoking during pregnancy is a combination of smoking during 1^st^ and 3^rd^ trimester. The numbers (n) in the adjusted analyses are slightly smaller due to missing values on covariates. REF: Randomly selected mothers and their children (reference group). | | | | | | |  |

**Table S5. Smoking during pregnancy and odds of child overweight (IOTF criteria) at age 7 years, adjusted for maternal transmitted GRS, in the combined reference group (REF) and groups of mothers with overweight and their children (MO-OW).**

|  | |  | Crude analysis | Adjusted^1^ | Adjusted^2^ | Adjusted^3^ | Adjusted^4^ | |
| --- | --- | --- | --- | --- | --- | --- | --- | --- |
|  | | **n** | OR (95% CI) | OR (95% CI) | OR (95% CI) | OR (95% CI) | OR (95% CI) | |
|  | |  |  |  |  |  |  | |
| **Any smoking during pregnancy^5^** | | 904 |  |  |  |  |  | |
| No | | 705 | 1.00 | 1.00 | 1.00 | 1.00 | 1.00 | |
| Yes | | 201 | 2.04 (1.39; 2.99) | 2.03 (1.36; 3.03) | 2.03 (1.38; 3.00) | 2.04 (1.37; 3.06) | 1.99 (1.33; 3.00) | |
|  | |  |  |  |  |  |  | |
| **Smoking 1^st^ trimester** | | 904 |  |  |  |  |  | |
| No smoking | | 705 | 1.00 | 1.00 | 1.00 | 1.00 | 1.00 | |
| 1-10 cigarettes per day | | 109 | 1.69 (1.02; 2.08) | 1.82 (1.07; 3.09) | 1.71 (1.03; 2.87) | 1.84 (1.08; 3.14) | 1.79 (1.04; 3.07) | |
| 11+ cigarettes per day | | 90 | 2.62 (1.59; 4.31) | 2.40 (1.43; 4.02) | 2.54 (1.54; 4.21) | 2.40 (1.43; 4.03) | 2.37 (1.40; 4.01) | |
|  | |  |  |  |  |  |  | |
| **Smoking 3^rd^ trimester** | | 850 |  |  |  |  |  | |
| No smoking | | 741 | 1.00 | 1.00 | 1.00 | 1.00 | 1.00 | |
| 1-10 cigarettes per day | | 50 | 2.68 (1.41; 5.07) | 2.68 (1.38; 5.21) | 2.59 (1.35; 4.98) | 2.62 (1.33; 5.15) | 2.49 (1.26; 4.93) | |
| 11+ cigarettes per day | | 59 | 2.68 (1.48; 4.83) | 2.42 (1.32; 4.44) | 2.80 (1.54; 5.10) | 2.57 (1.40; 4.74) | 2.55 (1.37; 4.75) | |
|  | 1. Adjusted for maternal BMI z-score, 2. Adjusted for maternal transmitted GRS, 3. Adjusted for maternal BMI and transmitted GRS, 4. Adjusted for maternal BMI, transmitted GRS and socioeconomic position, 5. Any smoking during pregnancy is a combination of smoking during 1^st^ and 3^rd^ trimester. The numbers (n) in the adjusted analyses are slightly smaller due to missing values on covariates. REF: Randomly selected mothers and their children (reference group); MO-OW: Mothers with overweight and their children (mothers with overweight group). | | | | | | |  |

**Table S6. Smoking during pregnancy and odds of child overweight (IOTF criteria) at age 7 years, adjusted for maternal non-transmitted GRS, in the combined reference group (REF) and groups of mothers with overweight and their children (MO-OW).**

|  | |  | Crude analysis | Adjusted^1^ | Adjusted^2^ | Adjusted^3^ | Adjusted^4^ |  |
| --- | --- | --- | --- | --- | --- | --- | --- | --- |
|  | | **n** | OR (95% CI) | OR (95% CI) | OR (95% CI) | OR (95% CI) | OR (95% CI) |  |
|  | |  |  |  |  |  |  |  |
| **Any smoking during pregnancy^5^** | | 904 |  |  |  |  |  |  |
| No | | 705 | 1.00 | 1.00 | 1.00 | 1.00 | 1.00 |  |
| Yes | | 201 | 2.04 (1.39; 2.99) | 2.03 (1.36; 3.03) | 2.0 (1.38; 2.98) | 2.04 (1.37; 3.04) | 1.99 (1.33; 2.98) |  |
|  | |  |  |  |  |  |  |  |
| **Smoking 1^st^ trimester** | | 904 |  |  |  |  |  |  |
| No smoking | | 705 | 1.00 | 1.00 | 1.00 | 1.00 | 1.00 |  |
| 1-10 cigarettes per day | | 109 | 1.69 (1.02; 2.08) | 1.82 (1.07; 3.09) | 1.68 (1.01; 2.93) | 1.83 (1.07; 3.10) | 1.77 (1.04; 3.02) |  |
| 11+ cigarettes per day | | 90 | 2.62 (1.59; 4.31) | 2.40 (1.43; 4.02) | 2.61 (1.59; 4.29) | 2.41 (1.44; 4.04) | 2.38 (1.41; 4.02) |  |
|  | |  |  |  |  |  |  |  |
| **Smoking 3^rd^ trimester** | | 850 |  |  |  |  |  |  |
| No smoking | | 741 | 1.00 | 1.00 | 1.00 | 1.00 | 1.00 |  |
| 1-10 cigarettes per day | | 50 | 2.68 (1.41; 5.07) | 2.68 (1.38; 5.21) | 2.64 (1.39; 5.01) | 2.73 (1.39; 5.29) | 2.59 (1.32; 5.08) |  |
| 11+ cigarettes per day | | 59 | 2.68 (1.48; 4.83) | 2.42 (1.32; 4.44) | 2.66 (1.48; 4.80) | 2.43 (1.33; 4.46) | 2.39 (1.29; 4.44) |  |
|  | 1. Adjusted for maternal BMI z-score, 2. Adjusted for maternal non-transmitted GRS, 3. Adjusted for maternal BMI and non-transmitted GRS, 4. Adjusted for maternal BMI, non-transmitted GRS and socioeconomic position, 5. Any smoking during pregnancy is a combination of smoking during 1^st^ and 3^rd^ trimester. The numbers (n) in the adjusted analyses are slightly smaller due to missing values on covariates. REF: Randomly selected mothers and their children (reference group); MO-OW: Mothers with overweight and their children (mothers with overweight group). | | | | | | | |

**Table S7. Smoking during pregnancy and odds of children being sampled into the group with overweight (CH-OW), adjusted for maternal transmitted GRS, based on the combined REF group and the CH-OW group.**

|  | |  | Crude analysis | Adjusted^1^ | Adjusted^2^ | Adjusted^3^ | Adjusted^4^ | |
| --- | --- | --- | --- | --- | --- | --- | --- | --- |
|  | | **n** | OR (95% CI) | OR (95% CI) | OR (95% CI) | OR (95% CI) | OR (95% CI) | |
| **Any smoking during pregnancy^5^** | | 1,257 |  |  |  |  |  | |
| No | | 904 | 1.00 | 1.00 | 1.00 | 1.00 | 1.00 | |
| Yes | | 353 | 1.66 (1.28; 2.15) | 1.59 (1.22; 2.08) | 1.64 (1.26; 2.13) | 1.59 (1.21; 2.08) | 1.47 (1.11; 1.94) | |
|  | |  |  |  |  |  |  | |
| **Smoking 1^st^ trimester** | | 1,253 |  |  |  |  |  | |
| No smoking | | 901 | 1.00 | 1.00 | 1.00 | 1.00 | 1.00 | |
| 1-10 cigarettes per day | | 185 | 1.30 (0.94; 1.80) | 1.26 (0.90; 1.77) | 1.29 (0.93; 1.79) | 1.26 (0.90; 1.78) | 1.17 (0.83; 1.65) | |
| 11+ cigarettes per day | | 167 | 2.32 (1.59; 3.38) | 2.20 (1.51; 3.27) | 2.27 (1.56; 3.32) | 2.19 (1.48; 3.23) | 2.06 (1.38; 3.06) | |
|  | |  |  |  |  |  |  | |
| **Smoking 3^rd^ trimester** | | 1,212 |  |  |  |  |  | |
| No smoking | | 1,003 | 1.00 | 1.00 | 1.00 | 1.00 | 1.00 | |
| 1-10 cigarettes per day | | 90 | 1.63 (1.02; 2.60) | 1.52 (0.94; 2.47) | 1.67 (1.04; 2.69) | 1.57 (0.96; 2.55) | 1.44 (0.88; 2.36) | |
| 11+ cigarettes per day | | 119 | 2.73 (1.74; 4.40) | 2.80 (1.74; 4.53) | 2.67 (1.67; 4.26) | 2.71 (1.68; 4.39) | 2.48 (1.51; 4.07) | |
|  | 1. Adjusted for maternal BMI z-score, 2. Adjusted for maternal transmitted GRS, 3. Adjusted for maternal BMI and transmitted GRS, 4. Adjusted for maternal BMI, transmitted GRS and socioeconomic position, 5. Any smoking during pregnancy is a combination of smoking during 1^st^ and 3^rd^ trimester. The numbers (n) in the adjusted analyses are slightly smaller due to missing values on covariates. CH-OW: Children with overweight and their mothers (children with overweight group); REF: Randomly selected mothers and their children (reference group). | | | | | | |  |

**Table S8. Smoking during pregnancy and odds of children being sampled into the group with overweight (CH-OW), adjusted for maternal non-transmitted GRS, based on the combined REF group and the CH-OW group.**

|  | |  | Crude analysis | Adjusted^1^ | Adjusted^2^ | Adjusted^3^ | Adjusted^4^ | |
| --- | --- | --- | --- | --- | --- | --- | --- | --- |
|  | | **n** | OR (95% CI) | OR (95% CI) | OR (95% CI) | OR (95% CI) | OR (95% CI) | |
| **Any smoking during pregnancy^5^** | | 1,257 |  |  |  |  |  | |
| No | | 904 | 1.00 | 1.00 | 1.00 | 1.00 | 1.00 | |
| Yes | | 353 | 1.66 (1.28; 2.15) | 1.59 (1.22; 2.08) | 1.66 (1.28; 2.15) | 1.59 (1.21; 2.08) | 1.47 (1.12; 1.94) | |
|  | |  |  |  |  |  |  | |
| **Smoking 1^st^ trimester** | | 1,253 |  |  |  |  |  | |
| No smoking | | 901 | 1.00 | 1.00 | 1.00 | 1.00 | 1.00 | |
| 1-10 cigarettes per day | | 185 | 1.30 (0.94; 1.80) | 1.26 (0.90; 1.77) | 1.30 (0.94; 1.80) | 1.26 (0.90; 1.77) | 1.17 (0.83; 1.65) | |
| 11+ cigarettes per day | | 167 | 2.32 (1.59; 3.38) | 2.20 (1.51; 3.27) | 2.32 (1.59; 3.37) | 2.20 (1.50; 3.26) | 2.07 (1.39; 3.08) | |
|  | |  |  |  |  |  |  | |
| **Smoking 3^rd^ trimester** | | 1,212 |  |  |  |  |  | |
| No smoking | | 1,003 | 1.00 | 1.00 | 1.00 | 1.00 | 1.00 | |
| 1-10 cigarettes per day | | 90 | 1.63 (1.02; 2.60) | 1.52 (0.94; 2.47) | 1.63 (1.02; 2.60) | 1.56 (0.96; 2.53) | 1.44 (0.88; 2.35) | |
| 11+ cigarettes per day | | 119 | 2.73 (1.74; 4.40) | 2.80 (1.74; 4.53) | 2.76 (1.73; 4.40) | 2.76 (1.71; 4.46) | 2.53 (1.54; 4.15) | |
|  | 1. Adjusted for maternal BMI z-score, 2. Adjusted for maternal non-transmitted GRS, 3. Adjusted for maternal BMI and non-transmitted GRS, 4. Adjusted for maternal BMI, non-transmitted GRS and socioeconomic position, 5. Any smoking during pregnancy is a combination of smoking during 1^st^ and 3^rd^ trimester. The numbers (n) in the adjusted analyses are slightly smaller due to missing values on covariates. CH-OW: Children with overweight and their mothers (children with overweight group); REF: Randomly selected mothers and their children (reference group). | | | | | | |  |
